# Neuron-specific protein interactions of *Drosophila* CASK-β are revealed by mass spectrometry

**DOI:** 10.3389/fnmol.2014.00058

**Published:** 2014-06-30

**Authors:** Konark Mukherjee, Justin B. Slawson, Bethany L. Christmann, Leslie C. Griffith

**Affiliations:** Department of Biology, Volen Center for Complex Systems, National Center for Behavioral Genomics, Brandeis UniversityWaltham, MA, USA

**Keywords:** *Drosophila*, GAL4/UAS, mass spectrometry, MAGUK, CASK, immunoprecipitation

## Abstract

Modular scaffolding proteins are designed to have multiple interactors. CASK, a member of the membrane-associated guanylate kinase (MAGUK) superfamily, has been shown to have roles in many tissues, including neurons and epithelia. It is likely that the set of proteins it interacts with is different in each of these diverse tissues. In this study we asked if within the *Drosophila* central nervous system, there were neuron-specific sets of CASK-interacting proteins. A YFP-tagged CASK-β transgene was expressed in genetically defined subsets of neurons in the *Drosophila* brain known to be important for CASK function, and proteins present in an anti-GFP immunoprecipitation were identified by mass spectrometry. Each subset of neurons had a distinct set of interacting proteins, suggesting that CASK participates in multiple protein networks and that these networks may be different in different neuronal circuits. One common set of proteins was associated with mitochondria, and we show here that endogenous CASK-β co-purifies with mitochondria. We also determined CASK-β posttranslational modifications for one cell type, supporting the idea that this technique can be used to assess cell- and circuit-specific protein modifications as well as protein interaction networks.

## Introduction

CASK (also known as Camguk and Caki in *Drosophila*) is a member of the membrane-associated guanylate kinase (MAGUK) family of scaffolding proteins. CASK is present in many cell types, including most neurons (Martin and Ollo, 1996). In *Drosophila*, the *Cask* gene encodes two families of proteins from two independent transcriptional start sites. CASK-β proteins, which are homologous to mammalian CASK, have a CaMK domain followed by two L27's, a PDZ, an SH3 and a guanylate kinase domain. The other proteins are CASK-α's which have a short unique N-terminal sequence before the PDZ and lacks the CaMK and L27 domains. CASK-α's are more similar to mammalian p55/MPP1 and may have functionally distinct roles (Slawson et al., 2011).

Most previous work on CASK's nervous system function has been conducted on transheterozygote deficiency animals lacking both the CASK and MPP1-like proteins. Adult deficiency animals are infertile and have severe locomotor defects (Martin and Ollo, 1996). This is accompanied by long latency in the giant fiber pathway and increased spontaneous release at the flight muscle neuromuscular junction (Zordan et al., 2005). Larvae lacking both proteins have locomotor defects (Sun et al., 2009) and decreased FM dye loading/unloading at the neuromuscular junction, as well as reduced evoked and spontaneous current amplitudes (Chen and Featherstone, 2011).

To look specifically at the role of the CASK protein in *Drosophila*, we generated a mutant that lacked only CASK-β proteins but retained the ability to make the shorter MPP1-like proteins (Slawson et al., 2011). These animals are fertile and much healthier than transheterozygote deficiency animals. In adults, selective loss of the CASK-β protein produces locomotor (Slawson et al., 2011) and learning defects (Malik et al., 2013). These phenotypes map to separate neuronal subpopulations.

At the molecular level, CASK in *Drosophila* has been shown to regulate the Eag potassium channel (Marble et al., 2005), alter CaMKII autophosphorylation to decrease its calcium-responsiveness (Lu et al., 2003; Hodge et al., 2006) and to interact with neurexin to potentially regulate vesicle fusion (Sun et al., 2009). In mammals, CASK has been associated with both presynaptic and postsynaptic function (for review see Hsueh, 2009) and with signaling to the nucleus (Hsueh et al., 2000). CASK may also regulate CaMKII autophosphorylation and synaptic growth, phenotypes in glutamatergic motor neurons (Gillespie and Hodge, 2013). The complex roles of CASK in behavior and the multitude of binding partners in the literature suggest that CASK may have different roles in different neuronal circuits.

To test the idea that CASK may have neuron type-specific binding partners, we developed methods to purify CASK-β protein complexes from neuronal subpopulations and identify associated proteins by mass spectrometry. We find that CASK-β (from here on referred to as just CASK) isolated from different neuronal groups is associated with distinct, but overlapping sets of proteins. Analysis of these proteins confirms CASK's role in synaptic function, and identifies a novel role for CASK as a mitochondrially-associated protein.

## Materials and methods

### Fly husbandry

All flies were raised at 25°C in a 12:12 h light:dark cycle. Flies were fed standard cornmeal-dextrose agar media. CASK proteins (full-length wild type β isoform or mutants containing a deletion of either the L27 or CaMK domains) tagged with a C-terminal YFP were expressed in a cell-specific manner using the GAL4/UAS system (Brand and Perrimon, 1993). All experiments were done on a CASK-β null genetic background [*CASK^P18^* allele (Slawson et al., 2011)] to ensure that the only CASK-β present was the product of the tagged transgene. Briefly, animals carrying a UAS-CASK-YFP transgene (made from a cDNA encoding isoform B) were crossed to animals carrying GAL4's which expressed in specific cell types. The GAL4's used in this study were: C155-GAL4 (Genotype:*P{GawB}elavC155*, Bloomington stock 43351; chromosome X), all neurons (Lin et al., 1994); C164-GAL4 (Genotype:*P*{*GawB*}*C164*, Bloomington stock 33807), a subset of adult neurons including some dopaminergic neurons (Slawson et al., 2011; Slawson et al., in preparation); TH-GAL4, (Genotype:*P*{*ple-GAL4.F*}, Bloomington stock 8848; Chromosome 3), dopaminergic neurons (Friggi-Grelin et al., 2003); and DILP2-GAL4 (Genotype:*P*{*Ilp2-GAL4.R*}, Bloomington stock 37516; Chromosome 2), neurons of the pars intercerebralis which express *Drosophila* insulin-like peptide 2 (Corl et al., 2004).

### Cell fractionation

Mitochondrial and cytosol fractions were separated as described (Bahadorani et al., 2010). Briefly, 50 flies were gently crushed in 1 mL of chilled mitochondrial isolation medium (MIM: 250 mM sucrose, 10 mM Tris (pH 7.4), 0.15 mM MgCl_2_) and then spun twice at 1000 × g for 5 min at 4°C to remove debris. Crude cytosol was then spun at 13,000 × g for 5 min at 4°C. The pellet, containing the mitochondria, was washed once with MIM before immunoblotting. The cytosolic supernatant was also collected for immunoblotting.

### Immunoprecipitation

Approximately 100 fly heads, collected by sieving after freezing flies in liquid nitrogen, were homogenized in 250 μl chilled homogenizing buffer (20 mM Tris pH 7.2, and protease inhibitors). 250 μl of chilled solubilization buffer (20 mM Tris pH 7.2, 2 mM EDTA, 200 mM NaCl, 2% deoxycholic acid, 2% Triton-X-100, and protease inhibitors) was added to the homogenates. Proteins were solubilized by gentle rocking at 4°C for 2 h. The lysates were then centrifuged at 20,000 × g for 20 min to pellet undissolved material. Solubilized lysates were filtered through an 0.2 μm filter and incubated overnight at 4°C with Chemotrek GFP-TRAP™ beads (Allele Biotechnology, San Diego, CA.), which had been previously equilibrated with wash buffer (20 mM Tris pH 7.2, 100 mM NaCl, 1% deoxycholic acid, 1% Triton X-100, and protease inhibitors). Following this overnight incubation period, the beads were washed thrice with ice cold wash buffer, boiled in SDS sample buffer, and subjected to SDS-PAGE electrophoresis (see above). The gels were analyzed by either Coomassie staining (for mass spectrometry) or immunoblot. For analysis of posttranslational modifications by mass spectrometry, immunopreciptation reactions included 50 mM NaF, 1 mM sodium orthovanadate and 0.1 μM Okadaic acid in the buffer.

### Immunoblots

Samples were separated by 8% SDS-PAGE, transferred to nitrocellulose, and visualized on immunoblots. Bound secondary antibody (used at 1:5000) was detected via enzymatic assay using ECL detection reagents (Amersham) and visualized with film using a Kodak X-OMAT 2000A Developer. Primary antibodies used: anti-ATP synthase subunit beta (MitoSciences MS503, 1:1000); anti-CASK (gift of Gisela Wilson, 1:1000); anti-tubulin (Sigma T-5168, 1:1000).

### Mass spectrometry

LC-MS/MS was carried out at the Taplin Biological Mass Spectrometry Facility, Cell Biology Department, Harvard Medical School. Coomassie-stained bands were cut from SDS-PAGE into approximately 1 mm^3^ pieces. Gel pieces were then subjected to a modified in-gel trypsin digestion procedure (Shevchenko et al., 1996) directly or for analysis of posttranslational modifications, samples were reduced with 1 mM DTT for 30 min at 60°C and then alkylated with 5 mM iodoacetamide for 15 min in the dark at room temperature before the in-gel trypsin digestion. Gel pieces were washed and dehydrated with acetonitrile for 10 min followed by removal of acetonitrile and complete drying in a speed-vac. Gel pieces were rehydrated in 50 mM NH_4_HCO_3_ containing 12.5 ng/μl modified sequencing-grade trypsin (Promega, Madison, WI) at 4°C. After 45 min, the excess trypsin solution was removed and replaced with 50 mM NH_4_HCO_3_ to just cover the gel pieces. Samples were then placed at 37°C overnight. Peptides were recovered by removing the NH_4_HCO_3_ solution, followed by one wash with a solution containing 50% acetonitrile and 1% formic acid. The extracts were then dried in a speed-vac (~1 h). Samples were stored at 4°C until analysis.

On the day of analysis, samples were reconstituted in 5–10 μl of HPLC solvent A (2.5% acetonitrile, 0.1% formic acid). A nano-scale reverse-phase HPLC capillary column was created by packing 5 μm C18 spherical silica beads into a fused silica capillary (125 μm inner diameter × ~20 cm length) with a flame-drawn tip (Peng and Gygi, 2001). After equilibrating the column each sample was loaded onto the column via a Famos auto sampler (LC Packings, San Francisco CA). Peptides were eluted with increasing concentrations of solvent B (97.5% acetonitrile, 0.1% formic acid).

As peptides eluted they were subjected to electrospray ionization and then entered into an LTQ-Velos or LTQ-Orbitrap ion-trap mass spectrometer (ThermoFisher, San Jose, CA). Peptides were detected, isolated, and fragmented to produce a tandem mass spectrum of specific fragment ions for each peptide. Peptide sequences (and hence protein identity) were determined by matching protein databases or translated nucleotide databases with the acquired fragmentation pattern using the software program, Sequest (ThermoFisher, San Jose, CA) (Eng et al., 1994). Spectral matches were manually examined and multiple identified peptides per protein were required. For posttranslational modifications, mass units corresponding to phosphate or acetate were included in the database searches and peptides were manually inspected to ensure confidence.

### Analysis of interactors

The complete list of detected peptides, the % coverage and the number of peptides is shown in Supplemental Table 1. We applied two criteria to determine the set of interactors for each cell type. First, as a criterion for significance, we only considered proteins for which >4 unique peptides were detected. Second, as a criterion for specificity, we only considered proteins for which the CASK-YFP immunoprecipitation produced at least a 4-fold greater number of peptides than the control GFP immunoprecipitation. A list of proteins which met this criterion is provided for each GAL4 driver in Supplemental Table 2.

To determine functional groupings of interactors, the CG numbers of genes for which peptides were found were identified from FLYBASE and used as input in DroPNet (Renaud et al., 2012), a web-based interface for building networks (https://dropnet.gred-clermont.fr/DroPNet_project/index.faces). Networks were created inclusive of all the filters. Hierarchical network figures were generated and served as a template for appropriate placement of nodes and edges in Adobe Illustrator.

For generating the pathway description table, plain text files of the proteins co-precipitated with CASK were uploaded on R-Spider interface (http://www.bioprofiling.de/gene_list.html). Two separate analyses were taken into consideration, ProfCom (Antonov et al., 2008) and R-Spider (Antonov et al., 2010) using the Reactome and KEGG databases to determine biological pathways of the precipitated proteins.

## Results

### Recovery of CASK complexes from neuronal subpopulations

**Figure 1:**
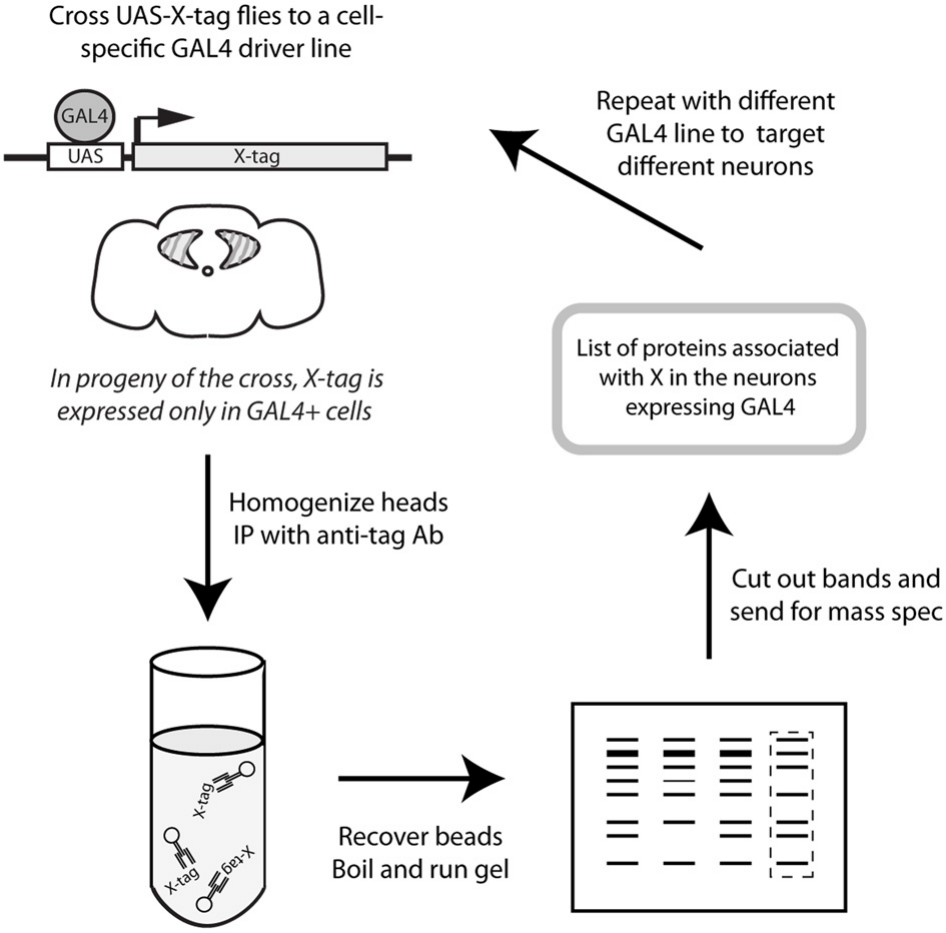
**Schematic representation of protocol**. CASK-YFP fusion proteins were expressed in a cell-specific manner using the GAL4/UAS system (Brand and Perrimon, 1993) on a CASK-β null background (Slawson et al., 2011) to ensure that the only CASK-β present was the product of the transgene. Adult heads were collected and homogenized, and CASK proteins were recovered with anti-GFP-conjugated beads. Eluted proteins were separated by SDS-PAGE and Coomassie-stained gels were sent for analysis by mass spectrometry to determine the proteins which associate with CASK in the cell type targeted by the specific GAL4 line.

In order to identify cell type-specific binding partners for CASK-β, we expressed YFP-tagged CASK-β in spatially restricted domains on a *CASK^P18^* null mutant background using the GAL4/UAS system (Brand and Perrimon, 1993). This ensured that the only source of full length CASK was the tagged transgene. The tagged proteins were recovered by immunoaffinity precipitation and their interacting proteins identified by mass spectrometry. Figure 1 shows a schematic of this strategy.

We chose to examine 4 neuronal populations defined by different GAL4 lines which we deemed potentially relevant for CASK's behavioral function. These lines were subsequently tested for their ability to rescue *CASK^P18^* phenotypes when wild type CASK was expressed. C155-GAL4 expresses in all neurons, while C164-GAL4 expresses in a subset of adult CNS neurons. Expression of CASK under control of either of these drivers can fully rescue the complex locomotor defects of the *CASK^P18^* mutant (Slawson et al., 2011). C164-GAL4 expresses in a subset of dopaminergic neurons (data not shown), as well as in mushroom bodies, pars intercerebralis, subesophageal ganglion, motor neurons and antennal lobes (Slawson et al., 2011). Behavioral experiments (Slawson et al., in preparation) dissecting the contribution of these regions to locomotor function concluded that TH-GAL4, which expresses in dopaminergic neurons, could rescue the initiation defects of the null mutant, consistent with a role for dopaminergic transmission in motor initiation in many species (Feany and Bender, 2000), so this line was also used for pull-down. Expression in the pars intercerebralis with DILP2-GAL4 did not rescue locomotion, so this line was chosen to serve as a non-motor-relevant control.

**Figure 2:**
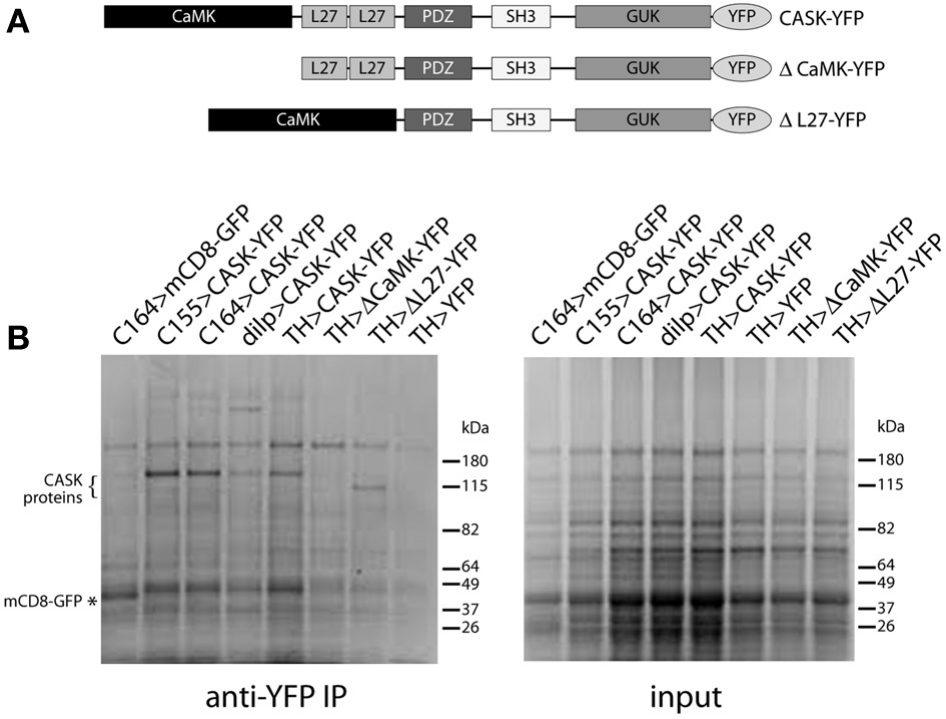
**Isolation of CASK-interacting proteins. (A)** Schematic of CASK-β proteins used in this study. **(B)** Proteins eluted from anti-GFP beads were separated by SDS-PAGE and visualized by Coomassie staining. Genotypes are indicated above each lane. Bands can be seen at the correct molecular weights for CASK-YFP and CASK-YFP deletion mutants (indicated by “{”) and for control mCD8-GFP (indicated by “*”). Identities of proteins were confirmed by immunoblotting (data not shown).

Domain organization of CASK transgenes used in these studies is shown in Figure 2A. Figure 2B shows Coomassie-stained gels of immunoaffinity purified complexes and starting material. CASK-YFP proteins and mCD8-GFP can be seen in the pull-downs, but not in the starting material. A large number of bands appear to be present in common, but there are also bands that are unique to each GAL4.

Supplemental Table 1 lists all proteins from which we recovered peptides. Significant interactors were defined as those proteins for which more than 4 peptides were present and which had a 4-fold or greater enrichment compared with a GFP control. Using these criteria, from the list of >1000 proteins detected in all the mass spec runs, we found 23 to be significantly associated with CASK in C155-GAL4 cells, 63 to be associated in C164-GAL4 cells, 84 to be associated in TH-GAL4 cells and 6 to be associated in DILP-GAL4 cells (Supplemental Table 2). In all data sets, CASK itself was recovered with ca. 20–30% coverage, indicating successful immunoprecipitation.

### Known CASK interactions are demonstrated in neuron-specific data sets with full length CASK

CASK has many binding partners (for review see, Hsueh, 2009). CASK was first identified as a neurexin binding protein (Hata et al., 1996) and subsequently found to be a member of a tripartite neuronal complex with Mint1 (x11) and Veli (Butz et al., 1998). Both neurexin and x11 (the fly Mint1 ortholog) were identified as significant interactors in the C155 and C164 samples and Veli was recovered in the C155 pull-down (Supplemental Table 1). CASK has also been shown to bind to and regulate CaMKII (Lu et al., 2003; Hodge et al., 2006) and this kinase was present in all samples except those derived from DILP2-GAL4 cells. The presence of known CASK-binding proteins in the pull-downs, but not the GFP control, suggests our technique can recover genuinely associated proteins.

There is also a significant amount of overlap in data sets from different neuronal subsets (Supplemental Table 3). While only 5 proteins other than CASK were found in significant amounts in all of the data sets, there were 22 that were found in 3 out of 4 of the neuron types. For this set of proteins the only cell type that did not have them was DILP2-GAL4. Whether this is due to the small number of these neurons and poor recovery of material compared to the other drivers, or to some difference in the function of CASK in peptidergic neuroendocrine cells remains to be determined.

### Analysis of CASK-associated proteins suggests that CASK is involved in both synaptic and metabolic functions

Pathway analysis of the significantly associated proteins for the C155, C164, and TH data sets is presented in Table 1.

**Table 1:** **Pathway analysis of associated proteins**.

**Process**	**Pathway ID**	**Description**	**# Of input genes**	**Input genes**	***p*-value**
**PATHWAYS ASSOCIATED WITH PROTEINS PULLED DOWN WITH C155-GAL4**					
Cellular process	GO:0007163	Establishment or maintenance of cell polarity	3	NRX CAKI VELI	0.01^*^
	RN00010	Glycolysis/gluconeogenesis	4	TPI PYK ENO CG8036	0.029^*^
	GO:0006468	Protein phosphorylation	3	CAMKII CAKI NINAC	0.295
**PATHWAYS ASSOCIATED WITH PROTEINS PULLED DOWN WITH C164-GAL4**					
Neurotransmission	GO:0016080	Synaptic vesicle targeting	3	NRX CAKI X11L	0.01^*^
	GO:0007269	Neurotransmitter secretion	6	CG1618 KHC ALPHA-ADAPTIN DAP160 CAKI X11L	0.075
Protein synthesis and transport	GO:0008104	Protein localization	5	NRX ALPHA-ADAPTIN DAP160 TER94 NINAC	0.01^*^
	GO:0006413	Translational initiation	5	EIF-4B EIF3-S9 EIF3-S10 CIF2 AGO2	0.025^*^
	GO:0006886	Intracellular protein transport	3	ALPHA-ADAPTIN NINAC ALPHACOP	0.325
Cellular process and tissue development	GO:0007163	Establishment or maintenance of cell polarity	3	NRX CAKI X11L	0.115
	GO:0007498	Mesoderm development	3	NK3 CHP PRM	0.325
**PATHWAYS ASSOCIATED WITH PROTEINS PULLED DOWN WITH TH-GAL4**					
Amino acid metabolism	RN00250	Alanine, aspartate and glutamate metabolism	6	GS2 SSADH CG1640 GOT1 CG7433 CG7145	0.01^*^
	RN00480	Glutathione metabolism	5	PGD IDH DIP-B ZW GCLM	0.175
	RN00350	Tyrosine metabolism	3	SSADH GOT1 ADH	0.175
Carbohydrate metabolism	RN00010	Glycolysis/gluconeogenesis	6	PGI TPI PYK PGK ADH ENO	0.025^*^
	RN00030	Pentose phosphate pathway	4	PGD CG8036 PGI ZW	0.035^*^
	RN00020	Citrate cycle (TCA cycle)	4	IDH ATPCL ACON KDN	0.165
	RN00500	Starch and sucrose metabolism	4	PGI UGP CG33138 GLYP	0.305
	RN00562	Inositol phosphate metabolism	3	TPI CG9391 INOS	0.305
Cellular homeostasis	GO:0055114	Oxidation reduction	16	SOD2 IDH SSADH CG31548 PGD CG6084 TRXR CG7145 GPO GCLM SU(R) PDH CG3523 ZW GDH ADH	
	GO:0045454	Cell redox homeostasis	6	PDI PRX5 ERP60 TRXR JAFRAC1 CG1837	0.065
Cellular process	GO:0015992	Proton transport	3	VHA55 VHA68 VHA26	0.285
System process	GO:0007611	Learning or memory	3	CAMKII GCLM CHER	0.315

*Associated proteins for each GAL4 pull-down were analyzed against the Reactome and KEGG databases using ProfCom (Antonov et al., 2008) and R-Spider (Antonov et al., 2010). A minimum of 5 genes was required for a pathway to be picked up. P-value is an estimate of the probability to infer the same size model from random gene list of the same size*.

* *denotes pathways that are represented significantly (P ≤ 0.05)*.

The small number of significant interactors in the DILP2 data set prevented meaningful pathway analysis for this line. In all 3 other neuronal sets, CASK has significant interactions with metabolic proteins. This is especially prominent in the TH data set, where many mitochondrial proteins are found associated with CASK. In the C155 and C164 data sets, a role for CASK in subcellular localization is suggested by the presence of proteins involved in formation of subcellular complexes.

CASK is also associated with a number of proteins involved in neurotransmitter release (Supplemental Table 2). While the stringency of the pathway analysis only renders this statistically significant for C164, Comatose (comt), the fly NSF homolog, is a significant hit in all but the DILP2 data set, and x11 and neurexin are present in both C164 and C155. Synapsin (Syn) was found in TH cells, but not other neuron types. Hsc70-4, a protein known to be involved in calcium-dependent neurotransmitter release in *Drosophila* (Bronk et al., 2001) was also present in the TH data set. This association with vesicle release complexes is consistent with synaptic phenotypes reported in *Drosophila CASK* mutants (Zordan et al., 2005; Sun et al., 2009; Chen and Featherstone, 2011).

### CASK is part of several protein interaction networks

**Figure 3:**
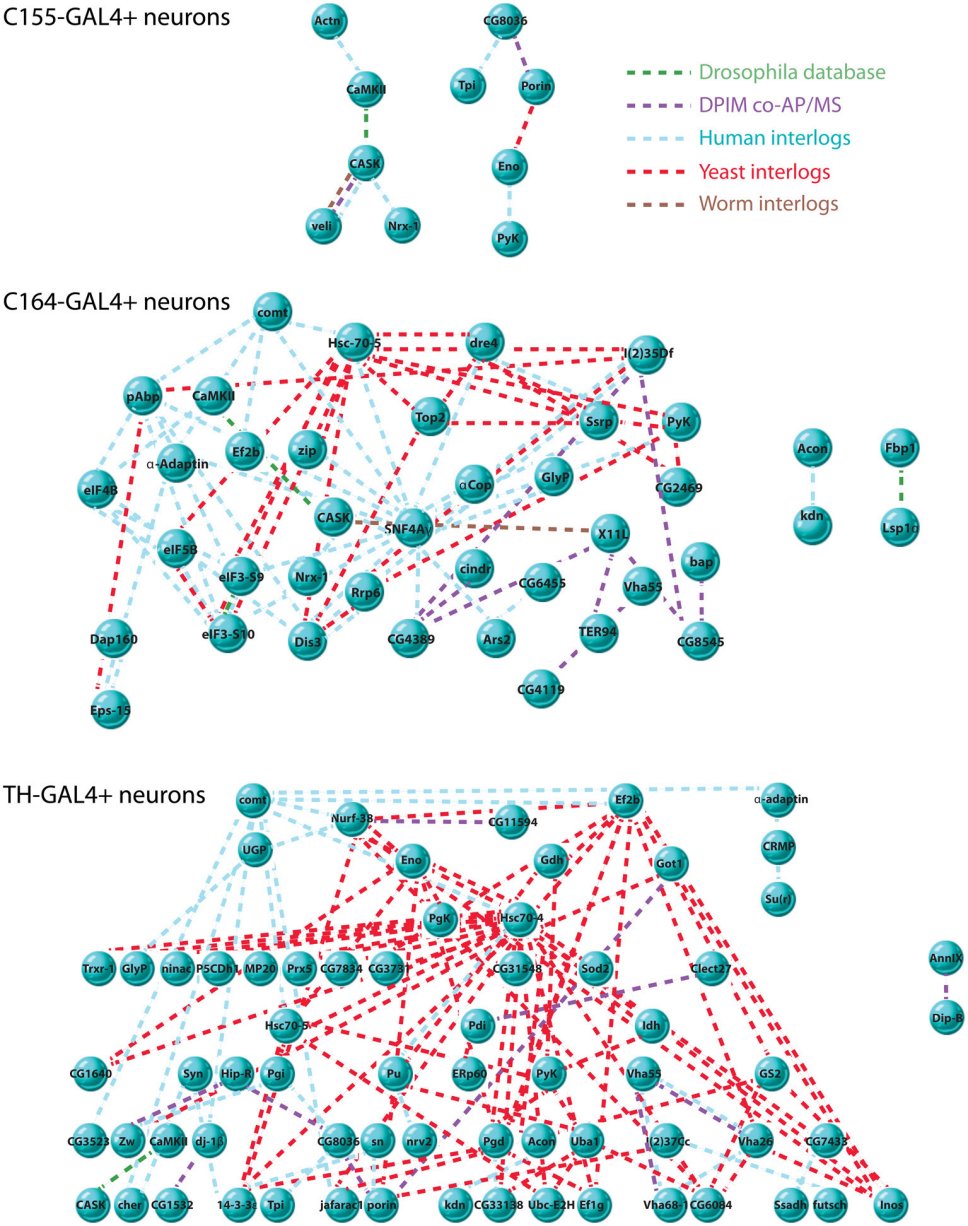
**Interaction networks of CASK-associated proteins**. Interaction networks were detected using DroPNet (Renaud et al., 2012), a web-based interface for building networks. Proteins found in each cell type were loaded into DroPNet and searched against *Drosophila*, Yeast, Human, and Worm protein interaction databases. Origin of each interaction is color-coded: *Drosophila* databases, green, *Drosophila* Protein Interaction Mapping project, purple, Human interlogs, blue, Yeast interlogs, red, and Worm interlogs, brown. Top: there were 10 interactions in the C155-GAL4 data set (23 proteins), describing 2 networks. 13 members of the data set showed no interactions and are not shown. The average number of interactions on 10 random gene lists of the same size was 0.2. Middle: there were 82 total interactions in the C164-GAL4 data set (63 proteins), describing 3 networks. 23 members of the data set showed no interactions and are not shown. The average number of interactions on 10 random gene lists of the same size was 1.5. Bottom: there were 122 interactions in the TH-GAL4 data set (84 proteins) describing 2 networks. 17 members of the data set showed no interactions and are not shown. The average number of interactions on 10 random gene lists of the same size was 1.4.

To further understand the nature of the relationships among the significantly associated proteins we recovered, we asked if there were physical interactions between the members of each data set that might define networks or larger complexes. Using DroPNet, we ran the data sets against protein interaction databases from fly, human, worm and yeast. There were several statistically significant networks uncovered for C155, C164, and TH, but again the DILP2 data set was too small to yield results. Figure 3 shows the networks.

It is notable that the C155 networks are much less complex than those found in TH and C164 neurons. Since C155 is a panneuronal driver, it is sampling a very heterogeneous pool of neuron types compared to the more restricted C164 and TH drivers. This might suggest that these two drivers are picking up cell type-specific interaction networks that are diluted when pull-down of CASK is performed from all neurons. The extent and interconnectedness of the C164 and TH neurons also suggests that some of the proteins in the pull-downs might not directly interact with CASK, but rather are brought down as part of a larger protein complex. Proteins that did not have known interactions with other CASK-associated proteins are not shown in the figure and may therefore reflect proteins for which direct interactions occur, although this would have to be validated.

### CASK is associated with mitochondria

**Figure 4:**
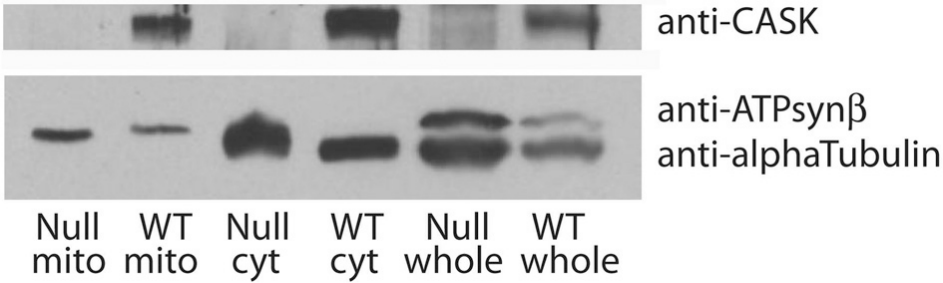
**CASK is associated with mitochondria**. Debris-cleared fly head extract (whole) was centrifuged to separate mitochondria (mito) from cytosol (cyt). Purity of samples was assessed by immunoblotting with anti-alpha Tubulin, a marker for cytosol and anti-ATP synthaseβ subunit, a marker for mitochondria. Samples from wild type (WT) and *CASK^P18^* mutant flies (null) were analyzed by immunoblotting. Upper panel shows CASK associated with both cytosolic and mitochondrial fractions in wild type fly heads.

A major feature of the groups of proteins associated with CASK is their involvement in metabolic processes. In particular, CASK appears to associate with a number of mitochondrial proteins, including DJ-1(dj-1β), a mitochondrial stability factor involved in Parkinson's disease (Chang et al., 2014). To determine whether this reflected the presence of CASK in or bound to mitochondria, we purified this organelle by differential centrifugation and immunoblotted the fractions for CASK, tubulin (as a marker for cytosol), and ATP synthaseβ subunit (as a marker for mitochondria). Figure 4 demonstrates that CASK is present in both cytoplasmic and mitochondrial fractions. Similar purification of mitochondria from *CASK^P18^* null animals shows no CASK band, demonstrating the specificity of the antibody.

### The CASK N-terminal domains have specific interactions

Previous work in our lab had shown that the CaMK and L27 domains in the N-terminal of the full length CASK-β protein were required for its role in locomotion, since loss of that protein, in the context of normal levels of the CASK-α MPP1-like protein, caused disruption of locomotor activity (Slawson et al., 2011). Motor initiation defects could be rescued by expression of full length CASK-β by either TH-GAL4 (Slawson et al., 2011), but not with expression of forms of CASK-β lacking either the CaMK or L27 domains (Slawson et al., in preparation). To see if loss of these domains changed the binding of particular associated proteins, we expressed the mutant proteins under control of TH-GAL4 and compared the recovered peptides to the data set of the 101 significantly associated proteins obtained for full length CASK.

The first immediately interesting observation was that there were no proteins whose recovery was unique to the mutant forms or was increased significantly (>2-fold) by loss of either of the domains. This implies that neither of these domains is inhibiting binding of ligands to other CASK domains (for schematic of domain organization see Figure 2A).

There were, however, a small number of proteins whose binding was substantially (≥2-fold) decreased by loss of one or both N-terminals domains, suggesting that these proteins are specifically interacting with the missing domains. Table 2 lists proteins lost from pull-downs of mutant CASK.

**Table 2:** **Domain-specific CASK interactions in dopaminergic neurons**.

**Decreased in ΔL27**		**Decreased in ΔCaMK**		**Decreased in both**	
**Gene**	**Function**	**Gene**	**Function**	**Gene**	**Function**
CG2947	Hsc binding protein	Idh	Isocitrate dehydrogenase	AnxB9	Annexin
Vha26	vacuolar H+ ATPase subunit	Pgk	Phosphoglycerokinase	CG3244	Lectin-like
		CASK	MAGUK	prtp	Thioredoxin
		Pdi	Protein disulfide isomerase	Pyk	pyruvate kinase
		cher	Cheerio, actin-binding		
		Prx5	Periredoxin		
		Syn	Synapsin		
		Vha68-1	Vacuolar H+ ATPase subunit		
		Uba1	Ubiquitin/SUMO transferase		
		GlyP	Glycogen phosphorylase		
		UGP	UTP–glucose-1-phosphate uridylyltransferase		
		Dip-B	Dipeptidase		
		CG3523-RA	Thioesterase		

*CASK proteins were expressed in dopaminergic neurons under control of TH-GAL4. There were no proteins found to be enriched by greater than 2-fold over full length CASK for either deletion mutant. Significant hits (≥4 peptides and either absence from GFP control pull-down or enrichment 4-fold) were calculated for CASK-YFP missing both L27 domains (ΔL27) and for CASK-YFP missing the CaMK domain (ΔCaMK). To be considered decreased, a protein had to have a peptide ratio ≤ 0.5 mutant/full length. Of the 101 proteins found to be associated with full length CASK in TH-GAL4 cells, only 19 were decreased by loss of N-terminal domains*.

The L27 domain had only 2 specific interactors, whereas the CaMK domain had 13. These included synapsin (Syn) and cheerio (cher), proteins involved in synaptic function and plasticity. The decrease in the number of CASK peptides recovered in the ΔCaMK pull-downs likely reflects a lack of stability of the truncated protein (see Figure 2B). An interesting point with this mutant is that there is still association of CaMKII indicating that there are likely binding sites for the kinase other than the CaMK domain. This is consistent with the data in Lu et al. (2003) that suggested interactions of purified CaMKII with multiple domain of CASK. There were also 4 proteins whose recovery was reduced in both mutants, suggesting they either may have binding sites that span both domains or that these domains interact to form a unique binding surface that is disrupted when one of the domains is deleted. Overall, the recovery of only 19 of 84 associated proteins changed, suggesting that the other 65 significantly associated proteins interact with domains common to CASK-β and the CASK-α isoforms.

### CASK is posttranslationally modified in C164-GAL4 neurons

Posttranslational modification of eukaryotic proteins is an effective way of regulating their function (Mann and Jensen, 2003). Posttranslational modification may alter protein-protein interactions, protein trafficking, enzymatic function and protein turnover (Seo and Lee, 2004). A challenge to proteomic experiments is to map these modifications accurately. Importantly, posttranslational modification can vary significantly in different cell types, which may contribute to cell-type specific diversification of protein functions (Dickerson and Mains, 1990; Christensen et al., 2007). Most proteomic experiments do not address these cell-specific differences, since proteins are typically precipitated only from whole organ lysates. Since we can express CASK in cell-specific manner using different GAL4 driver lines, we decided to test the possibility of detecting cell type-specific posttranslational modifications using this methodology.

We isolated transgenic CASK-YFP from the C164-GAL4 cells using anti-GFP in the presence of phosphatase inhibitors and high levels of EDTA to inhibit kinases and acetyltransferases. In this experiment we were able to acquire ~80% coverage of CASK-YFP. We detected three peptides which were posttranslationally modified (Table 3).

**Table 3:** **Posttranslational modification of CASK**.

**Modification**	***Drosophila* CASK**	**Human CASK**
Acetylation	YLA**K**@HNAIFDTLDVVTYEEVVK	YLA**K**HNAVFDQLDLVTYEEVVK
Phosphorylation	DVYGEEALRV**T#**PPPMVPYLNGDELDNVEGGELQHVTR	EVYSDEALRV**T**PPPTSPYLNGDSPESANGGMDMENVTR
Phosphorylation	HNAIFD**T#**LDVVTYEEVVK	HNAVFD**Q**LDLVTYEEVVK

*CASK-YFP was expressed under control of C164-GAL4, and then immunoprecipitated and analyzed by mass spectrometry for posttranslational modifications. The type of modification is indicated in the leftmost column, while the location of modifications is shown in the second column. @ indicates acetylation of the preceding residue, while # indicates phosphorylation of the preceding residue. The human CASK sequence of the homologous region is shown in the third column. A single acetylation site was found at aa 394 and this lysine is conserved in human CASK. Two phosphothreonines were detected at positions 149 and 402. Only the threonine at position 149 is conserved in the human protein. The bold values signify the post-translationally modified residues in Drosophila CASK and the corresponding residues in human CASK*.

Two of these peptides were phosphorylated at threonine residues, while one of the peptides was acetylated on a lysine residue. CASK is an atypical kinase and known to be a phosphoprotein (Mukherjee et al., 2008, 2010), and it is also a substrate for CDK5 (Samuels et al., 2007).

The phosphorylation sites on mammalian CASK have been mapped in COS cells (Samuels et al., 2007). Surprisingly, in our experiments we did not see phosphorylation of the cognate residues of the *Drosophila* CASK, indicating that the phosphorylation in this type of neuronal cell may be different than in renal cells. One of the threonines phosphorylated in *Drosophila* CASK is conserved in almost all CASK orthologs, indicating that that phosphorylation may also be evolutionarily conserved.

Protein acetylation is a key posttranslational modification, regulating various aspect of cell biology. Target proteins that are acetylated include histones, nuclear transcription factors, cytoskeletal proteins and proteins involved in cellular metabolism (Choudhary et al., 2009). Surprisingly, we discovered that a lysine residue in CASK is acetylated. This lysine is conserved in human CASK, suggesting that it could be a conserved modification in mammalian CASK as well. Our results clearly provide a proof of principle for using this methodology to map posttranslational modifications in a cell specific manner *in vivo*.

## Discussion

### Uncovering cell- or circuit-specific protein functions

The limited coding capacity of the genome has resulted in animals using the same gene products in different ways at discrete times and places in the organism. This has been especially common in the brain where many genes implicated in early development (e.g., Notch) have been shown to have critical adult-specific functions in behavior (Presente et al., 2004). It is likely, however, that the diversity of utilization of particular gene products may go even deeper. Complex organs like the brain are composed of numerous cell types organized into circuits which carry out specific brain functions. To achieve a full understanding of how these circuits function, it is necessary to understand the exact roles of the molecules present in the neurons. For proteins like CASK, which have many potential interactors, their role in a particular circuit cannot be understood without information about which of their many partners they engage in that neuron type. This serves to emphasize the need to develop methods to isolate cell-specific protein interactions and modifications if we are to understand the function of molecules in their native contexts. The technique that we have used to examine this issue for CASK, a MAGUK scaffolding protein with numerous known interactions, may prove to be one which could be used to investigate this problem for other proteins. It may also prove useful for examining changes in posttranslational modification or protein partners in a single cell type after some behavioral manipulation (learning, sleep deprivation, etc.).

### Potential pitfalls

The successful use of our method makes several assumptions. The first is that there will not be “mixing” of complexes/associations after cell lysis. Many protein-protein interactions are quite stable and exchange is most likely a problem for interactions that are low affinity or have fast off rates. The inclusion of salt and detergents can select for stronger interactions, but there is a risk of missing interesting low affinity partners. The fact that we see unique proteins for the different cell types or groups of cell types suggests that under our conditions we have been able to maintain specificity for a subset of protein partners. In contrast, for proteins that are common between all samples, interpretation is less clear. Either the strength of the interaction needs to be investigated to rule out potential for exchange in the lysate or some functional evidence of interaction from all the cell types needs to be obtained.

A second assumption that is made is that the amount of the tagged protein is in the physiologically relevant range in the cell. Overexpression could drive artifactual association with proteins that would not normally form complexes with the experimental molecule. With CASK, we expressed the tagged protein on a null background and we know that the amount of protein expressed is sufficient to rescue locomotor defects without inducing locomotor hyperactivity associated with overexpression of CASK on a wild type genetic background (Slawson et al., 2011; Slawson et al., in preparation). This behavioral titration provides confidence that the amount of CASK in our experiments is appropriate.

### Neuron specific functions of CASK: regulation of neurotransmitter release?

One of the first postulated roles for CASK in the nervous system was regulation of neurotransmitter release. While the presence of known interacting proteins (e.g., neurexin, Mint1/x11) and the pathway analysis (Table 1) support this role, the absence of particular proteins from a subset of the neuron types may also be informative. It has been shown for many proteins, including CASK, that binding sites can have multiple potential partners and that competition between partners can result in loss of particular interactions. In the case of Mint1/x11, which we see in C164- and C155-GAL4 pull-downs, but not in TH- or DILP2-GAL4 cells, previous work has shown that Caskin can disrupt its interaction with CASK (Tabuchi et al., 2002). Whether some similar competition with another ligand is behind the failure of Mint1/x11 to be detected in DILP2 or TH cells remains to be explored. Similarly, neurexin interacts with CASK via its PDZ domain and the absence of neurexin in DILP2 and TH cells may indicate either absence of neurexin in these cells or a steric competition. The differences in transmitter release-related interactors in different cell types may point to participation of CASK in cell-specific release complexes.

### Neuron-specific functions of CASK: metabolism?

The pull-down of a large number of metabolic and mitochondrial enzymes, particularly from dopaminergic cells, suggests that CASK may have a previously unappreciated role in regulation of cellular energy stores. This is supported by our finding that CASK is physically associated with mitochondria. The fact that CASK mutants show Parkinsonian-like defects in locomotor initiation (Slawson et al., 2011) which can be rescued by expression of full length CASK in dopaminergic cells (Slawson et al., in preparation) indicates that this protein has a unique role in this cell type. In mammalian models of Parkinson's disease, defects in energy balance and oxidative stress are believed to be at the heart of the pathological process. *Dj-1*, a gene implicated in familial Parkinson's has been shown to be a mitochondrial stability factor and we show here it is a CASK-associated protein.

### Neuron specific functions of CASK: transcriptional regulation?

In the sets of cell-specific associated proteins we obtained, it is notable how different the DILP2 data set is from the other neuron types. DILP cells are neuroendocrine in nature, and likely have a different vesicle release machinery, which may account for the lack of presynaptic proteins involved in small transmitter release. But what is most remarkable is the presence of histones. CASK is known to be capable of translocating to the nucleus under some conditions (Hsueh et al., 2000) and to interact with CINAP, a nucleosome-modifying protein (Wang et al., 2004). The identification of histones in the pull-downs from DILP cells may suggest that in these cells CASK has a significant nuclear presence, different from its role in dopaminergic or other non-peptidergic neurons.

### Concluding remarks

In this study we demonstrate that CASK, a MAGUK scaffolding protein which has been shown to interact with many different protein binding partners, participates in distinct protein complexes in different neuronal populations. The method we have used, expression of tagged protein in a subset of neurons to identify by mass spectrometry its cell- or circuit-specific interacting partners, is one that could be utilized in a variety of systems to understand the specific role of a protein in a specialized neuron type. This technique has the potential to give fine-grained information on protein interactions that cannot be obtained from identification of complexes from whole brain where there is a mixture of many cell types. This kind of analysis will allow us to build hypotheses about cell-specific molecular functions that will advance our understanding of behavior.

### Conflict of interest statement

The authors declare that the research was conducted in the absence of any commercial or financial relationships that could be construed as a potential conflict of interest.
