# Supplementary material for: Neuron-specific protein interactions of Drosophila CASK-β are revealed by mass spectrometry
Source: Front Mol Neurosci. 2014 Jun 30;7:58. doi: 10.3389/fnmol.2014.00058 (PMC4075472; doi:10.3389/fnmol.2014.00058)
Supplement: Supplementary file 3 [file DataSheet3.DOCX]

**Supplemental Table 3. Shared hits across neuron types**

| **Gene Name** | **Protein Name** | **C155** | **C164** | **Dilp2** | **TH** |
| --- | --- | --- | --- | --- | --- |
|  | | *4 out of 4* | | | |
| ninaC | NINAC_DROME | + | + | + | + |
| chp-RA | B7FNR3_DROME | + | + | + | + |
| GlyP | A4UZZ4_DROME | + | + | + | + |
| Pu-RB | C6TP60_DROME | + | + | + | + |
| Ef2b | A4V101_DROME | + | + | + | + |
|  | | *3 out of 4* | | | |
| 14-3-3epsilon | 1433E_DROME | + | 0 | + | + |
| CaMKII | A4V133_DROME A4V134_DROME D1YSG7_DROME | + | + | 0 | + |
| CG3731 | Q9VFF0_DROME | + | + | 0 | + |
| CG7145 | Q8T3P0_DROME | + | + | 0 | + |
| CG8036 | Q7KSU6_DROME | + | + | 0 | + |
| Eno | ENO_DROME | + | + | 0 | + |
| Fas1 | A8JR25_DROME | + | + | 0 | + |
| kdn-RA | C6TP50_DROME | + | + | 0 | + |
| PyK | KPYK_DROME | + | + | 0 | + |
| Arr1 | ARRA_DROME | + | + | 0 | + |
| beta-Spec | SPTCB_DROME | + | + | 0 | + |
| comt | NSF1_DROME | + | + | 0 | + |
| Adh | ADH_DROME | + | + | 0 | + |
| Fbp1 | FBP1_DROME | + | + | 0 | + |
| Gs2-RC | C8VV58_DROME | + | + | 0 | + |
| Lsp2 | LSP2_DROME | + | + | 0 | + |
| Pgd | 6PGD_DROME | + | + | 0 | + |
| proPo-A3 | PRPA3_DROME | + | + | 0 | + |
| Scp1 | O16157_DROME | + | + | 0 | + |
| Thiolase | O77466_DROME | + | + | 0 | + |
| UGP | A5XCL5_DROME | + | + | 0 | + |
| Vha55 | VATB_DROME | + | + | 0 | + |
| Yp3 | VIT3_DROME | + | + | 0 | + |
|  | | *2 out of 4* | | | |
| norpA | PIPA_DROME | + | 0 | + | 0 |
| 5PtaseI | B7Z0Q1_DROME | + | + | 0 | 0 |
| Actn | ACTN_DROME | + | 0 | 0 | + |
| CG2907 | Q8IGG7_DROME | + | 0 | 0 | + |
| Neurexin | A5HBQ4_DROME | + | + | 0 | 0 |
| porin | VDAC_DROME | + | 0 | 0 | + |
| sif | A8JNL4_DROME | + | + | 0 | 0 |
| Tpi | TPIS_DROME | + | 0 | 0 | + |
| Yp1 | Q29QE3_DROME | + | + | 0 | 0 |
| Acon | A8DZ21_DROME  Q9VIE8_DROME | 0 | + | 0 | + |
| α-Adaptin | AP2A_DROME | 0 | + | 0 | + |
| Bap | Q24253_DROME | 0 | + | 0 | + |
| CG1618 | A9YH29_DROME | 0 | + | 0 | + |
| CG4389 | Q8IPE8_DROME | 0 | + | 0 | + |
| CG6455 | IMMT_DROME | 0 | + | 0 | + |
| eIF-4B | Q7PLL1_DROME | 0 | + | 0 | + |
| Lsp1alpha | LSP1A_DROME | + | + | 0 | 0 |
| Lsp1beta | LSP1B_DROME | + | + | 0 | 0 |
| Pgk | PGK_DROME | + | 0 | 0 | + |
| Ssadh | Q9VBP6_DROME | + | 0 | 0 | + |
| t | Q9W369_DROME | + | 0 | 0 | + |
| TER94-RC | D0IQG4_DROME | + | + | 0 | 0 |
| Tudor-SN | Q8T0F1_DROME  Q9W0S7_DROME | 0 | + | 0 | + |
| Uba1 | O46111_DROME | 0 | + | 0 | + |
| Vha68-1 | Q8MLX7_DROME | 0 | + | 0 | + |
| X11L (mint1) | Q9GQQ6_DROME | + | + | 0 | 0 |
| dj-1beta | Q9VA37_DROME | + | 0 | 0 | + |
| Gpo-1 | Q7K569_DROME | 0 | + | 0 | + |
